# Combination Intensity Modulated Proton Therapy and Passive Scatter Boost for Rapidly Progressing Nasal Cavity Squamous Cell Carcinoma

**DOI:** 10.7759/cureus.1685

**Published:** 2017-09-14

**Authors:** Sweet Ping Ng, Ethan B Ludmir, Manuel A Oyervides, Richard Y Wu, Steve Frank, G Brandon Gunn

**Affiliations:** 1 Department of Radiation Oncology, The University of Texas MD Anderson Cancer Center

**Keywords:** proton, nasal cavity, radiotherapy

## Abstract

Cancers of the nasal cavity and septum are associated with poor prognosis and are usually treated with surgery followed by post-operative radiotherapy with or without concurrent chemotherapy. Definitive radiotherapy is used in cases where the tumor is unresectable, patient is unfit for surgery, and/or the patient declines surgical intervention. Here, we present a case of a patient, who for non-medical reasons, opted to have non-surgical management of his rapidly progressing nasal cavity tumor. He was successfully treated with concurrent chemoradiotherapy utilizing a combination of intensity modulated proton therapy (IMPT) with passive scatter boost to reduce dose to the adjacent critical neural structures. Post-treatment clinical examination and imaging demonstrated complete clinical and metabolic response at the primary site and neck. This case highlights the use of IMPT and passive scatter boost in combination to achieve delivery of therapeutic dose to nasal cavity tumor and neck whilst limiting dose to numerous adjacent organs-at-risk.

## Introduction

Cancers of the nasal cavity and septum are rare, accounting for less than 1% of head and neck mucosal cancers and are associated with poor prognosis [1]. These carcinomas are usually treated with surgery followed by post-operative radiotherapy with or without concurrent chemotherapy. Definitive radiotherapy has been used in cases in which the tumor is unresectable, patient is unfit for surgery, and/or the patient declines surgical intervention. Traditionally, definitive radiotherapy can be offered as a curative option in cases with small, anterior disease to avoid potentially disfiguring surgical resection. For more advanced cases, upfront surgical resection is usually recommended as it can be challenging to deliver definitive radiation doses to the primary tumor due to its close proximity to radiation-sensitive optic apparatus and other critical neural structures. Proton therapy has the physical advantage of depositing dose to the target area with minimal exit dose, compared to photon therapy [2-3]. With the development of intensity modulated proton therapy (IMPT), proton therapy can be delivered in a highly conformal fashion thereby reducing the low dose scatter to surrounding normal tissues such as the optic structures and the brain in a patient with nasal cavity tumor. Here, we present a case of rapidly progressing nasal cavity tumor successfully treated with IMPT and concurrent carboplatin.

## Case presentation

A 67-year-old Caucasian man, of good performance status, presented with a two-year history of a gradually enlarging left-sided nasal mass. He was a current smoker. The patient reported that over the past month prior to presentation, the mass grew rapidly and was causing intermittent epistaxis and pain within his left nose, prompting a visit to the doctor while he was overseas. A biopsy of the mass performed overseas revealed squamous cell carcinoma. Upon returning to the United States, the patient saw a surgeon who recommended upfront surgery including total rhinectomy with delayed reconstruction. For occupational/personal reasons, the patient presented to our institution for a second opinion, particularly interested in an ‘organ preservation’ treatment approach.

**Figure 1:**
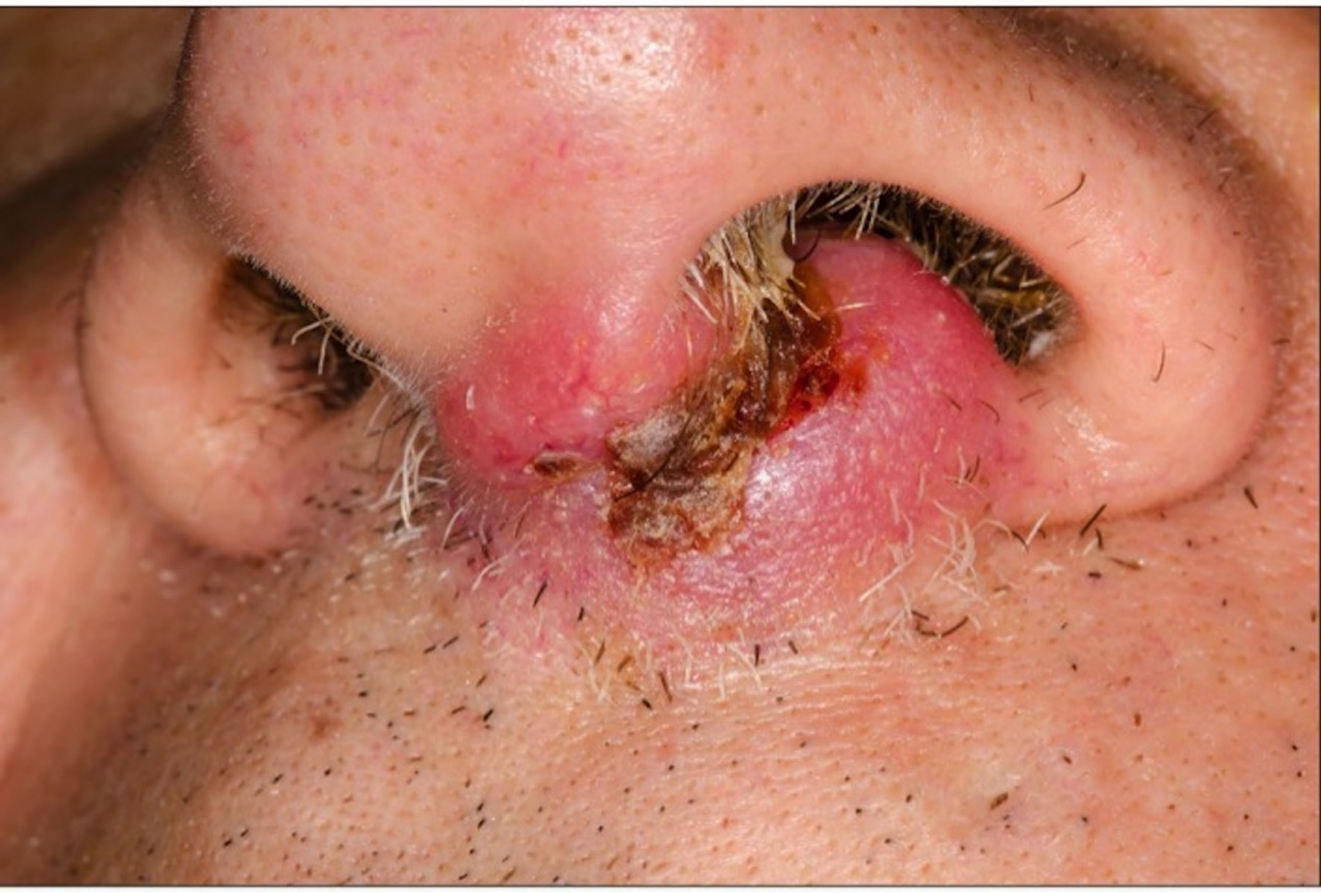
Left nasal mass at initial consultation. Clinical photograph depicting the nasal mass involving the nasal vestibule, columella, philtrum and the left nasal septum.

On initial consultation, the mass was ulcerated and involved the nasal vestibule, columella, philtrum and the left nasal septum (Figure 1). The left nasal ala was uninvolved. Palpation of the oral cavity revealed fullness within the upper gingivo-buccal sulcus at midline. The upper lip was involved from midline to the left side of the nasal vestibule. There were palpable bilateral submandibular masses of approximately 1 cm in size, which were mobile and non-tender. A fiberoptic nasopharyngolaryngoscopy showed an erythematous floor of the left nasal cavity extending to mid nasal cavity.

**Figure 2:**
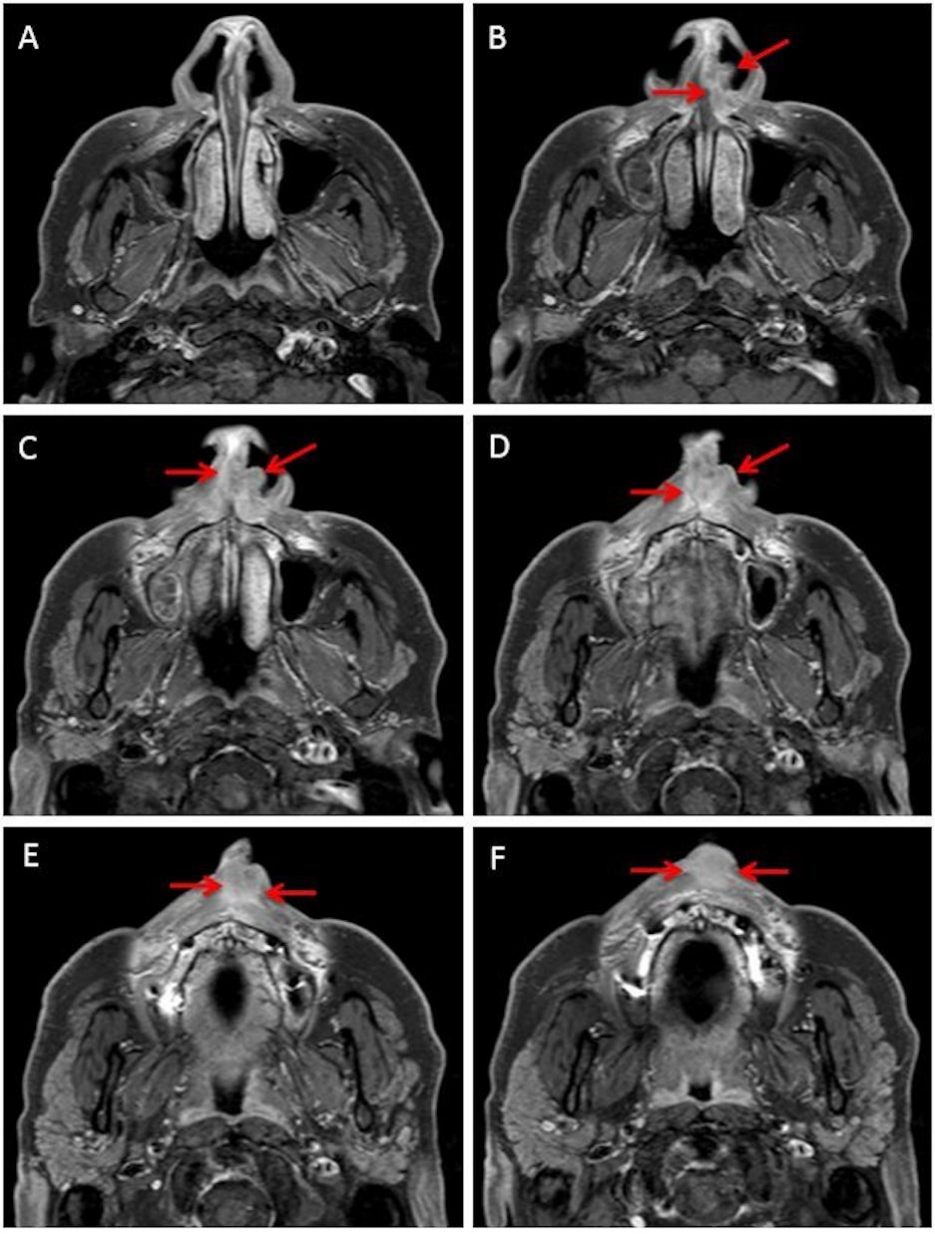
Serial axial slices of magnetic resonance imaging (MRI). Representative axial images of MRI. The extent of the tumor is illustrated with red arrows.

**Figure 3:**
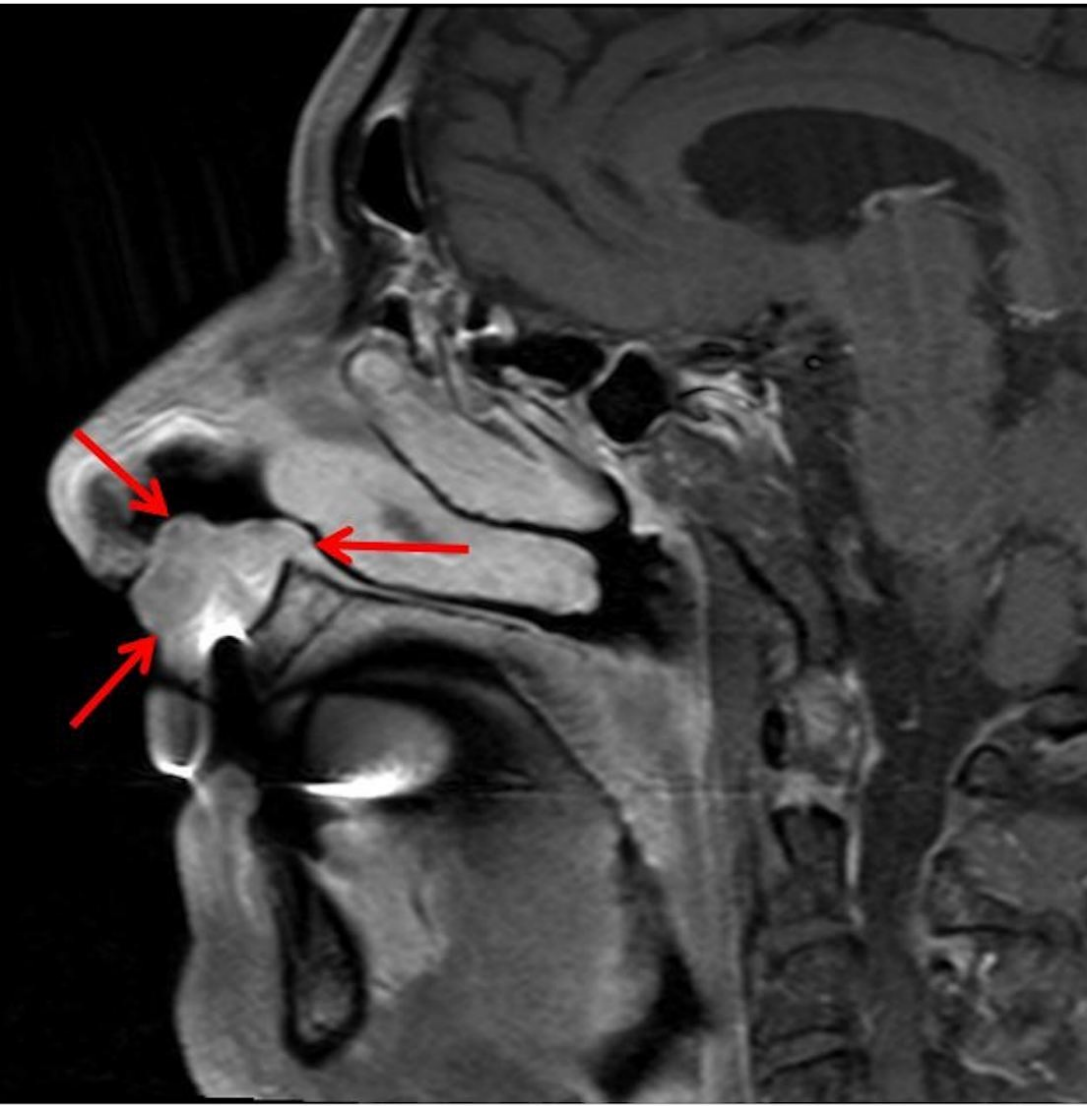
Sagittal slice of the MRI. Representative sagittal slice of MRI showing the extent of the tumor (red arrows). MRI: Magnetic resonance imaging

Magnetic resonance imaging (MRI) (Figures 2, 3) revealed a 1.3 x 2.9 x 2.2 cm mass over the left aspect of the anterior nasal septum extending inferiorly into the columella and philtrum.

**Figure 4:**
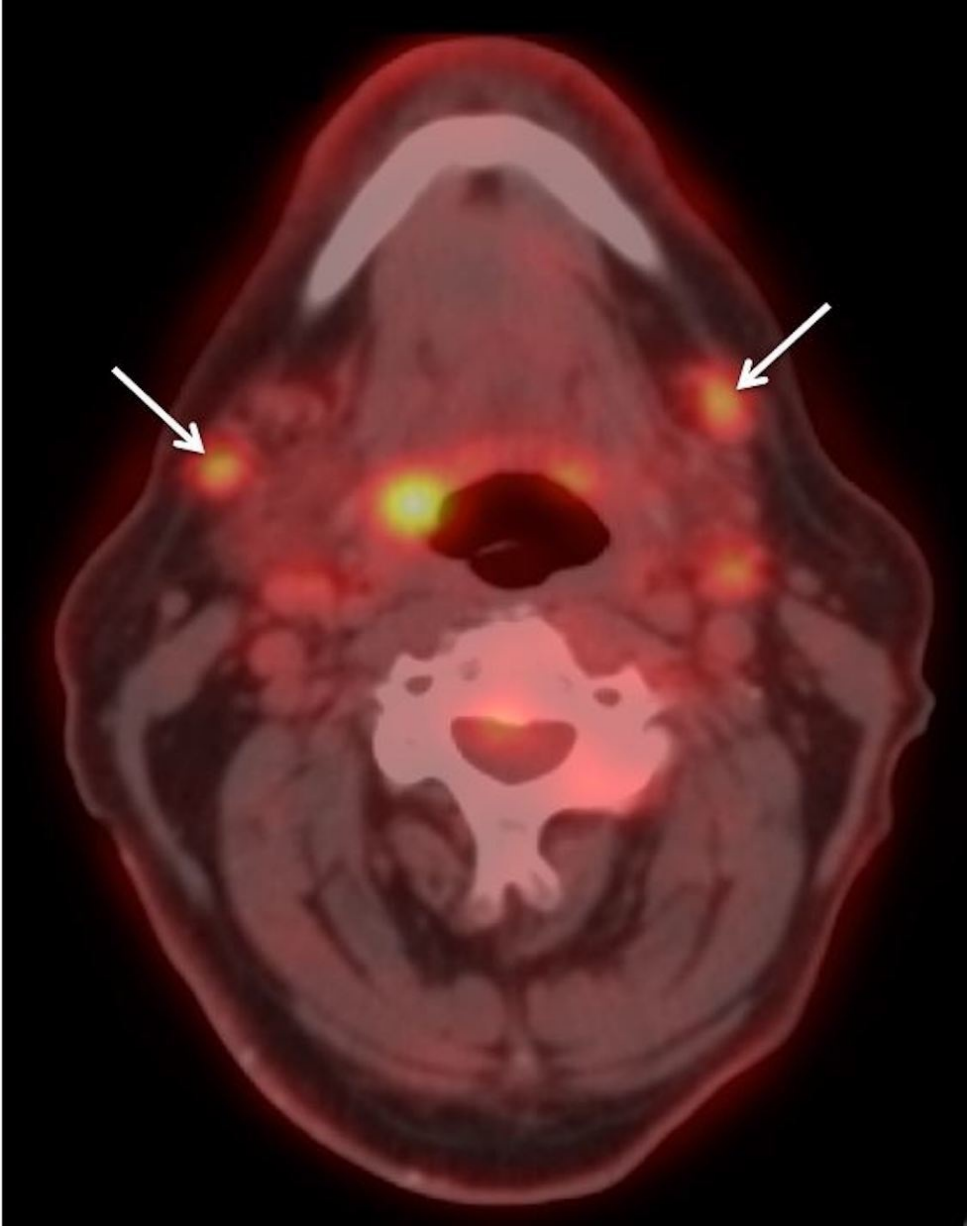
PET imaging. Representative PET image showing bilateral avid submandibular nodes (arrows). PET: Positron emission imaging

Positron emission tomography (PET) imaging demonstrated a hypermetabolic inferior anterior nasal septal and columellar mass with slight avidity of bilateral level IB (submandibular) nodes (Figure 4). There was no evidence of distant metastatic disease. A biopsy of the submandibular nodes revealed cells suspicious for squamous cell carcinoma. Overall, this tumor was staged cT2N2cM0 squamous cell carcinoma of the left nasal columella and cavity.

**Figure 5:**
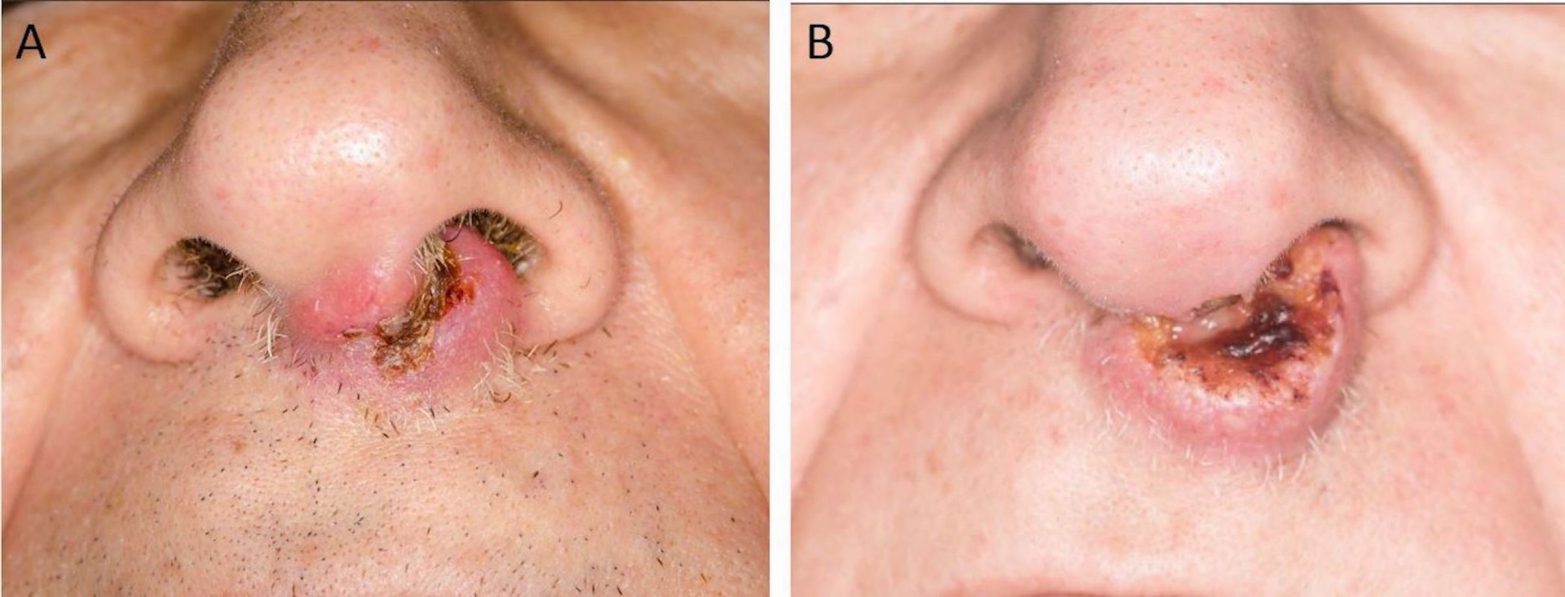
Clinical photographs showing progression of the nasal mass within a week before therapy. Image A – tumor at initial consultation. Image B – tumor noticeably larger one week after consultation and prior to induction chemotherapy.

His case was discussed at the multidisciplinary tumor board conference and the consensus was to recommend upfront surgery. Due to his occupation as a public figure, the patient declined surgery and opted for definitive chemoradiotherapy. During his initial week of evaluation and consultation at our institution, the patient had rapidly progressive disease with increasing pain and size of nasal mass over a week (Figure 5). Therefore, he was treated with one cycle of induction chemotherapy with carboplatin and paclitaxel to bridge his time between required dental extractions (pre-radiotherapy preparation) and start of radiotherapy. He was not a candidate for brachytherapy boost due to tumor proximity to bone and extension along the floor of the nose. Given the proximity of his tumor to critical neural and ophthalmologic structures he was treated with IMPT to a total dose of 70 Gray (Gy) radiobiologic equivalent (RBE) in 33 fractions. Treatment was accelerated initially to compensate for rapid tumor growth and was given over 6.5 weeks. He received concurrent weekly carboplatin.

**Figure 6:**
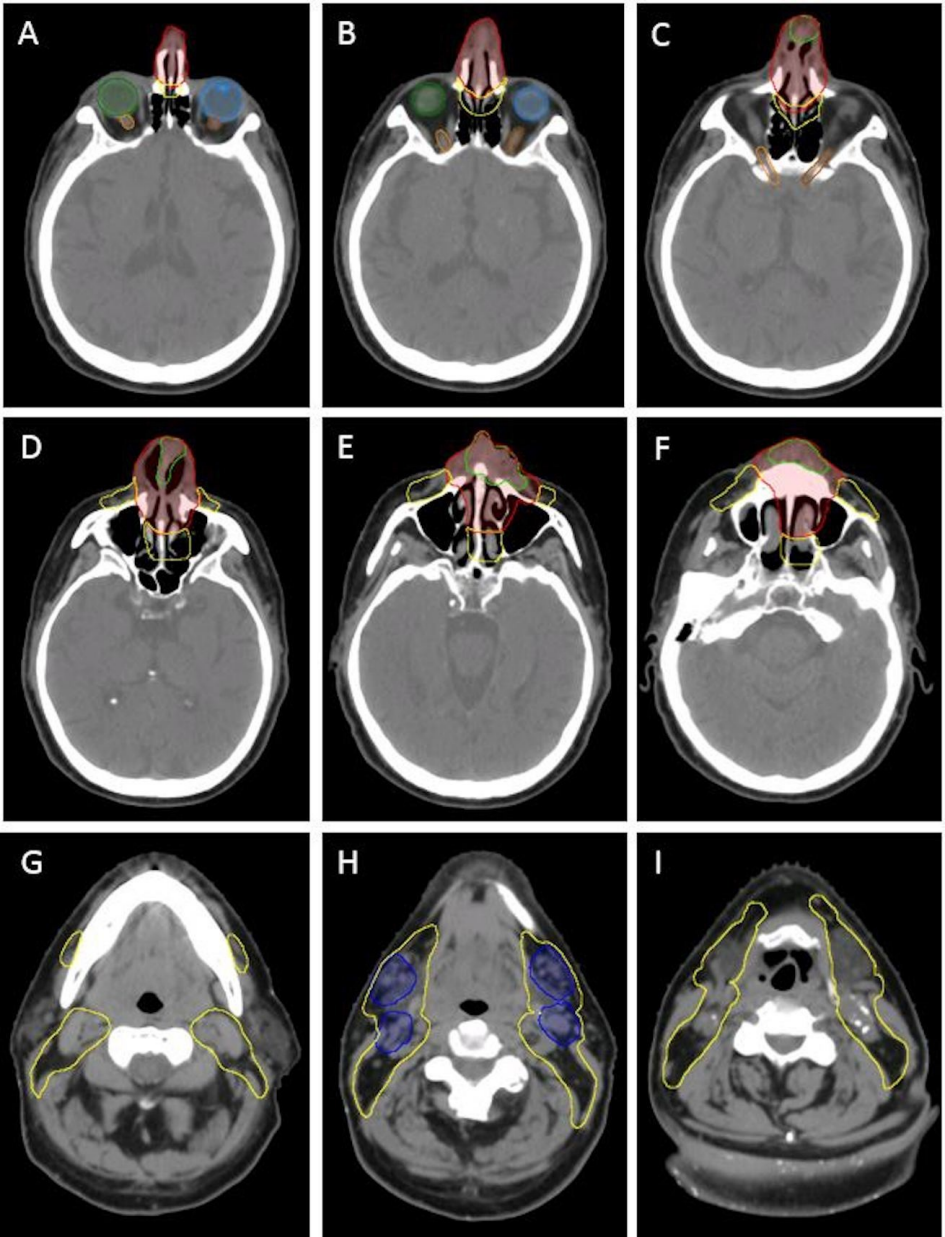
Simulation CT with delineated target (high, intermediate and low dose) regions. Images A–F show the primary tumor delineation and region of high dose in red and elective coverage in yellow. Images G–I show the involved nodal region (blue) to receive intermediate dose and elective coverage in yellow. Light green – gross tumor volume (GTV), red (primary tumor clinical target volume) to receive 70 Gy, dark blue (nodal disease clinical target volume) to receive 66 Gy, yellow – elective draining nodal regions to receive 50 Gy. Note the proximity of the high dose (red) contour to the optic apparatus: light blue – left eye, green – right eye, brown – left optic nerve, orange – right optic nerve. CT: Computed tomography

Computed tomography (CT) simulation was performed. A custom mouth-opening and tongue-depressing stent with bite block was fabricated to displace the oral tongue away from the palate/high dose target volumes. A customized posterior mold and thermoplastic mask were used. An MRI was also obtained in the radiation treatment position and MRI images were fused with planning CT images to aid with the target and organs-at-risk delineation. The primary gross tumor volume (GTV) and bilateral nodal GTVs, as well as multiple target dose levels were delineated (Figure 6). The primary tumor with margin was planned to 70 Gy RBE, the involved bilateral submandibular nodes with margin to 66 Gy RBE and elective nodal drainage regions (facial nodes and bilateral cervical levels I – IV) to 50 Gy RBE.

This was accomplished using a combination of active scanning beam IMPT (multi-field optimization, MFO) to 50 Gy RBE in 25 fractions to the nasal cavity and bilateral neck targets, followed by separate passive scatter boost fields to the primary tumor (20 Gy RBE in 10 fractions) and positive lymph nodes (16 Gy RBE in eight fractions). Custom apertures and compensators were used for the passive scatter boost fields to provide the greatest degree of lateral conformality and sparing of the anterior eyes, particularly the bilateral corneas.

**Figure 7:**
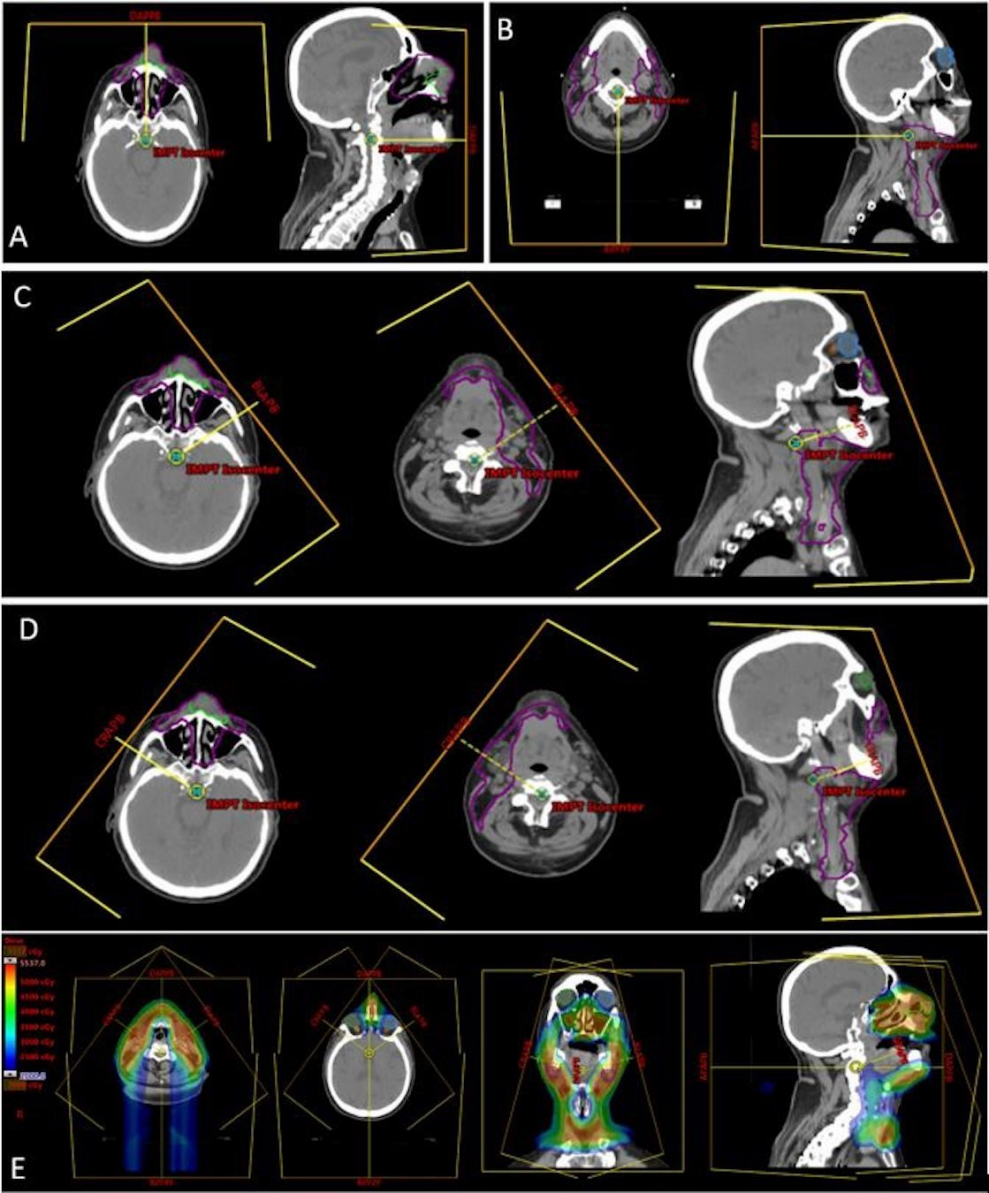
Representative images from the IMPT plan (see Table 1 for description). The purple contour line in each image indicates the beam specific target for each beam. IMPT: Intensity modulated proton therapy

**Figure 8:**
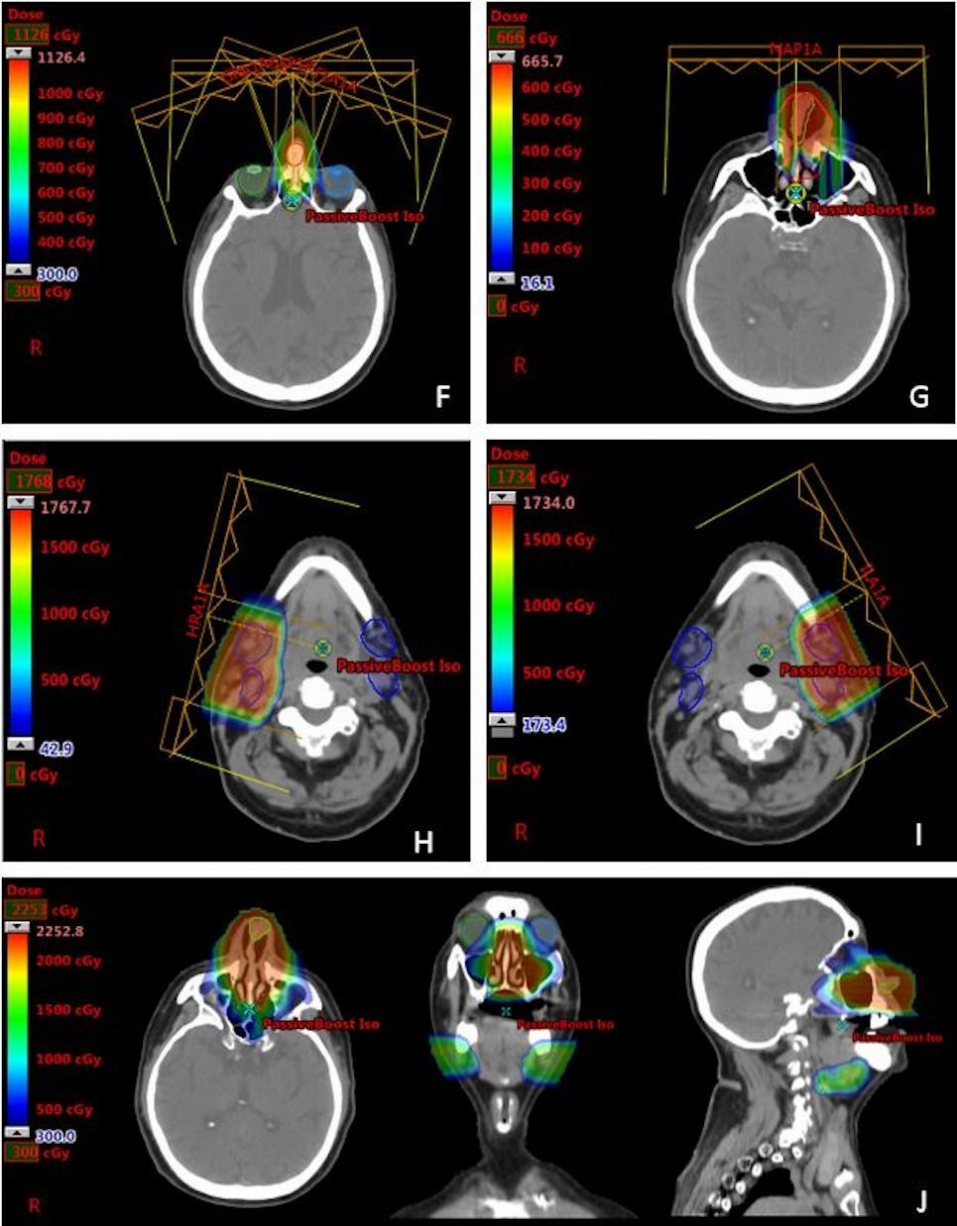
Representative images from the boost plan (see Table 1 for full description). F - nasal tumor boost fields (five fractions; three fields); G - nasal tumor boost (three fractions - single beam was used to reduce patient's time on the treatment table as he had claustrophobia); H - Right neck boost fields; I - Left neck boost field; J - sum of boost plans (left - axial, middle - coronal, right - sagittal).

**Figure 9:**
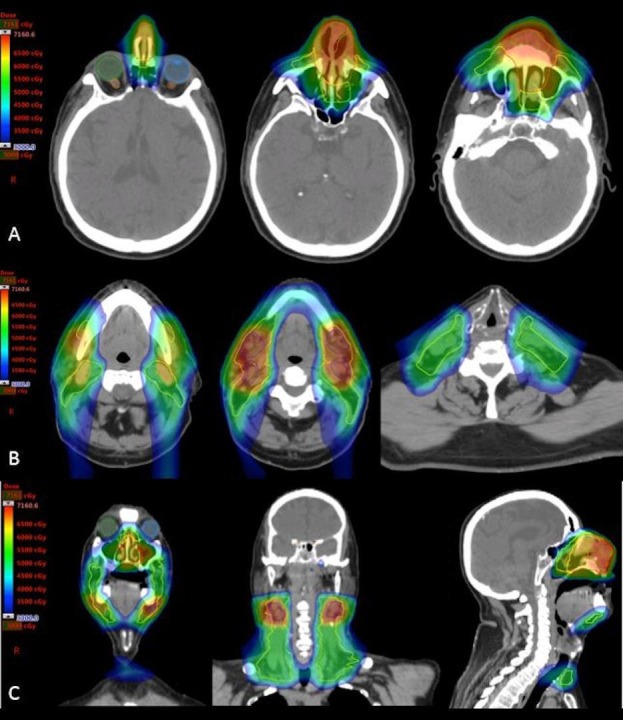
Representative images depicting overall combined dose distribution of the entire radiation treatment course. Note sparing of the optic apparatus, critical neural structures (optic nerves, brain, spinal cord) and oral cavity with IMPT with these structures receiving less than 30 Gy. Images in row A show the dose distribution within the primary tumor region. Images in row B show the dose distribution covering the involved and elective nodal regions. Images in row C show the combined IMPT and passive scatter boost treatment plan. IMPT: Intensity modulated proton therapy

Due to patient's difficulty with claustrophobia, the number of treatment fields was reduced as much as feasible (Table 1). To improve sparing of central uninvolved normal structures such as the spinal cord, oral cavity and larynx, a total of four beams were utilized and were planned to beam-specific targets for the IMPT plans (Figure 7). Additional attention was given to the region adjacent to the dental metal artifacts to ensure adequate target coverage and to reduce heterogeneities secondary to scattering effect. Furthermore, with the aim of reducing patient’s time on the treatment table, the boost to the nasal and bilateral submandibular nodal disease was planned with a single isocenter (Figure 8). The overall treatment plan is depicted in Figure 9. The patient had weekly verification CT simulation scans in the treatment position with immobilization devices to ensure satisfactory target dose coverage in the context of anatomic changes during treatment such as tumor regression, sinus cavity opacification/aeration variation, and/or patient weight loss. In addition, thermoluminescent dosimeters (TLDs) were placed over the tumor and nose during treatment and confirmed delivery of planned IMPT surface dose (within +/-5% for three TLDs).

**Table 1:** Proton delivery technique. Proton delivery technique and beam angles used to treat the patient at each phase with representative plan images (Figures 7, 8).

Phase	Technique	No. of Beams	Angles (in degrees)	Weighting	Reference figure
Nasal cavity and neck (50 Gy/ 25 fractions)	IMPT	4 beams Each beam was planned to a specific target	0	1.0	Figure 7 A
			180	1.0	Figure 7 B
			55	1.0	Figure 7 C
			305	1.0	Figure 7 D
		Plan sum			Figure 7 E
Boost (16–20 Gy/8–10 fractions)	Passive scattering	Nasal 3 beams (5 fractions)	0, 20, 340	0.3 each	Figure 8 F
		Nasal 1 beam (3 fractions)	0	1.0	Figure 8 G
		Right neck 1 beam	285	1.0	Figure 8 H
		Left neck 1 beam	60	1.0	Figure 8 I
		Plan sum			Figure 8 J

**Figure 10:**
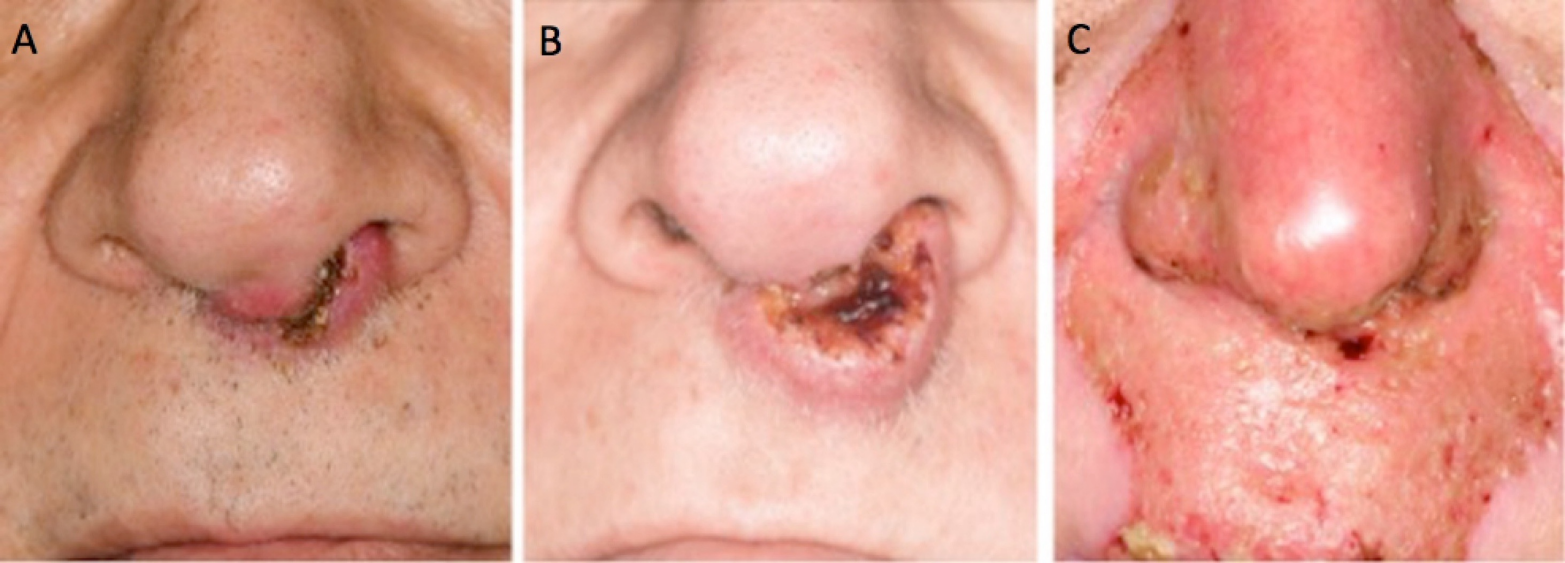
Nasal tumor at initial consultation (A), progression before therapy (B), and complete clinical response at the end of treatment (C).

During treatment, he developed Grade 3 dermatitis and oral mucositis requiring a five-day treatment break. With adequate pain management and topical treatments, he completed his course of treatment with accelerated (twice-daily) treatments in the final week to account for accelerated tumor cell repopulation during treatment break. Two weeks after the completion of radiotherapy, he was recovering well from the acute toxicities of treatment and the nasal mass had responded dramatically with no clinical evidence of disease (Figure 10).

Due to the aggressive nature of the disease at presentation, short interval restaging imaging and clinical examination was performed. PET imaging at five weeks post-treatment showed complete metabolic and radiographic response with mild fluorodeoxyglucose (FDG)-avidity within left submandibular node consistent with post-radiotherapy changes at this interval following treatment completion. Repeat PET imaging and clinical follow-up at three months post-radiotherapy showed complete metabolic and radiographic response. The patient had since returned to pre-morbid activities with no significant treatment-related toxicity.

## Discussion

In this case, a combination proton plan using IMPT (MFO) for the initial fields followed by separate passive scatter boost fields to the primary tumor and the involved left and right submandibular nodes provided excellent coverage of a geometrically complex and large target volume with sufficient sparing of the immediately adjacent and numerous critical and avoidance organs. His cutaneous reactions were consistent with desired therapeutic dose for this externally located tumor and resolved by three months after the treatment ended. Clinical tumor response was complete at the end of treatment and was metabolically complete at both five weeks and three months after treatment completion.

Overall, the annual incidence of sinonasal cavity cancers in the United States is approximately 2000 cases, consisting 3% of head and neck malignancies [4]. Squamous cell carcinomas are the most common histology of nasal cavity and paranasal sinuses cancers, and are associated with poor prognosis as they often present with advanced stage of disease [1].

There are no prospective randomized data to guide treatment recommendation in nasal cavity tumors. The current standard of care for advanced nasal cavity cancers is surgical resection followed by post-operative radiotherapy. The upfront surgical approach is usually preferred partly due to the difficulty to deliver a definitive dose of radiation to the tumor while respecting the dose tolerance of nearby normal structures including the optic apparatus, brain, and swallowing structures. Due to personal and functional concerns, our patient had opted for definitive radiotherapy with surgery reserved for salvage if needed.

Although there is no prospective level I evidence supporting the use of proton or photon radiotherapy in sinonasal cavity tumors, a meta-analysis by Patel, et al. [5] found that patients who received proton therapy had significantly better five-year disease-free survival (relative risk 1.44, 95% CI: 1.01 – 2.05, p = 0.045) and locoregional control (relative risk 1.26, 95% CI: 1.05 – 1.51, p = 0.011) than those who received photon therapy. This effect may be secondary to the theoretical higher relative biological effectiveness of protons compared to photons [6], thereby resulting in greater cell kill and possibly improved tumor control.

## Conclusions

In this case report, we highlighted the exceptional response of a rapidly progressing nasal cavity tumor to chemoradiotherapy. The use of IMPT and passive scatter in this case enabled the delivery of high dose of radiation to the nasal region whilst limiting dose to the nearby dose-sensitive orbital apparatus and neural structures.
